# A simplified but robust method for the isolation of avian and mammalian muscle satellite cells

**DOI:** 10.1186/1471-2121-13-16

**Published:** 2012-06-21

**Authors:** Belinda Baquero-Perez, Suresh V Kuchipudi, Rahul K Nelli, Kin-Chow Chang

**Affiliations:** 1 School of Veterinary Medicine and Science, University of Nottingham, Sutton Bonington Campus, College Road, Loughborough, Leicestershire, LE12 5RD, UK

**Keywords:** Muscle satellite cells, Primary skeletal muscle cultures, Immunocytochemistry, Desmin, Pax7, α-sarcomeric actin, Fusion index

## Abstract

**Background:**

Current methods of isolation of muscle satellite cells from different animal species are highly variable making inter-species comparisons problematic. This variation mainly stems from the use of different proteolytic enzymes to release the satellite cells from the muscle tissue (sometimes a single enzyme is used but often a combination of enzymes is preferred) and the different extracellular matrix proteins used to coat culture ware. In addition, isolation of satellite cells is frequently laborious and sometimes may require pre-plating of the cell preparation on uncoated flasks or Percoll centrifugation to remove contaminating fibroblasts. The methodology employed to isolate and culture satellite cells *in vitro* can critically determine the fusion of myoblasts into multi-nucleated myotubes. These terminally differentiated myotubes resemble mature myofibres in the muscle tissue *in vivo*, therefore optimal fusion is a keystone of *in vitro* muscle culture. Hence, a simple method of muscle satellite cell isolation and culture of different vertebrate species that can result in a high fusion rate is highly desirable.

**Results:**

We demonstrate here a relatively simple and rapid method of isolating highly enriched muscle satellite cells from different avian and mammalian species. In brief, muscle tissue was mechanically dissociated, digested with a single enzyme (pronase), triturated with a 10-ml pipette, filtered and directly plated onto collagen coated flasks. Following this method and after optimization of the cell culture conditions, excellent fusion rates were achieved in the duck, chicken, horse and cow (with more than 50% cell fusion), and to a lesser extent pig, pointing to pronase as a highly suitable enzyme to release satellite cells from muscle tissue.

**Conclusions:**

Our simplified method presents a quick and simple alternative to isolating highly enriched muscle satellite cell cultures which can subsequently rapidly differentiate into well developed primary myotubes. The use of the same isolation protocol allows better inter-species comparisons of muscle satellite cells. Of all the farm animal species investigated, harvested chicken muscle cells showed the highest percentage of muscle satellite cells, and equine muscle cells presented the highest fusion index, an impressive ≈ 77%. Porcine cells displayed the lowest amount of satellite cells but still achieved a modest fusion rate of ≈ 41%.

## Background

Skeletal muscle cells are complex multi-nucleated structures that specialize in contractile movement. During the prenatal period of muscle development, embryonic myoblasts initially increase in number (hyperplasia) by mitosis, later align and fuse into large post-mitotic multi-nucleated cells, myotubes, which further mature into contractile myofibres. Subsequently, post-natal/post-hatch development of muscle cells is characterised by an increase in the size of myofibres (hypertrophy), accomplished in part by the addition of myoblasts to the myofibre sarcolemma [1]. Myoblasts are derived from muscle satellite cells (a population of resident stem cells), which reside between the basement membrane and the myofibre sarcolemma [2]. When the muscle tissue is subjected to injury, stretch or exercise, muscle satellite cells are activated, divide and differentiate into myoblasts [3-5]. Similar to the embryological development of muscle, myoblasts undergo repeated mitosis and fuse into a pre-existing myotube or form new myotubes.

Several methods have been described for the isolation and *in vitro* culture of muscle satellite cells from skeletal muscles to study various aspects of skeletal muscle biology. An early method to isolate muscle satellite cells from rat skeletal muscle was described in 1974 [6]. Since then, a wide variety of modifications have been carried out to isolate satellite cells from other species such as human [7], chicken [8], turkey [9], horse [10], cow [11], sheep [12] pig [13] and rabbit [14]. In essence, all these methods involve common or similar steps which are mechanical mincing of muscle, followed by enzymatic digestion to release satellite cells, and differential centrifugation to separate the satellite cells from muscle debris. Following centrifugation, freed cells are plated onto culture flask coated with muscle extracellular matrix proteins such as collagen type I, gelatine, laminin or fibronectin. If further enrichment of muscle satellite cells is required, cells could be pre-plated on uncoated culture plates to remove faster adhering fibroblasts or centrifuged through Percoll gradients [8] which is more time consuming and labour intensive.

Primary cultures derived from skeletal muscles are often mixed populations of muscle cells and non-myogenic cells such as adipocytes, immune cells (e.g. macrophages) and particularly fibroblasts. Primary cell cultures are an invaluable tool because they are obtained directly from a normal animal, do not contain tumour cells unlike immortal cell lines and more closely represent *in vivo* muscle cells. Primary skeletal muscle cultures have been used in various studies involving development of new medical applications [15-18], better understanding of muscle physiology and even production of *in vitro* meat [19,20].

Most of the approaches that have been used to date for the isolation and culture of satellite cells do not usually include characterization of types of cells in culture. Studies conducted with an excessively heterogeneous cell population may not accurately provide valid insights into the biology of skeletal muscles *in vitro*. Identification of primary muscle cells can be achieved either by morphological features of the cells or by analysing the expression of muscle-specific genes. While the multi-nucleated myotubes in culture could be readily identified, a significant proportion of mononucleated cells cannot be distinguished from non-myogenic cells. Hence, identification of muscle-specific marker proteins by immunocytochemistry is valuable in distinguishing myogenic and non-myogenic cells in culture.

In addition, most of the methods described for the isolation of primary cultures of skeletal muscle are time consuming and complex. There are no standardized methods that could be used to isolate satellite muscle cells across a variety of different species. Hence, there is a need for a simple, rapid and reproducible method that will generate a high yield of muscle satellite cells from different species. We report here on the development of a simplified protocol that can be used for the rapid isolation and identification of primary muscle cells from chicken, duck, horse, cow and pig skeletal muscles. We found this method to be cost efficient and yield high number of primary muscle cells that readily fuse to form myotubes in all the species tested.

## Results and discussion

**Figure 1:**
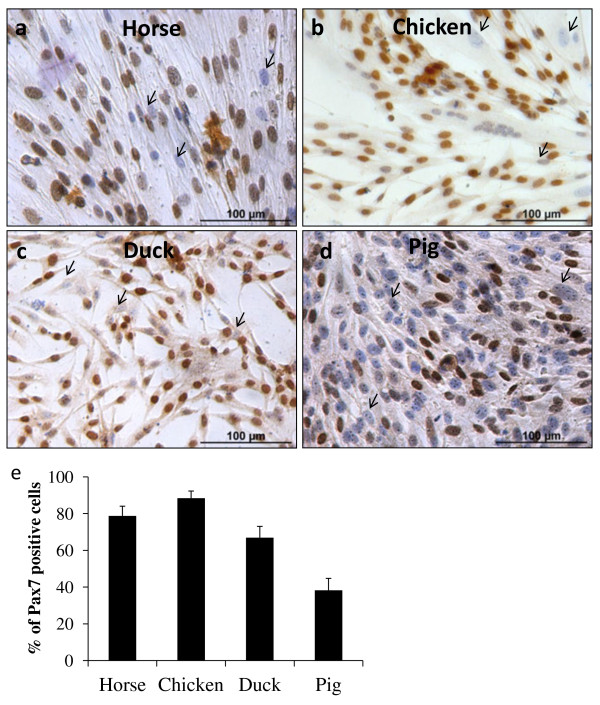
**Pax7 expression appears to be exclusively confined to myoblasts of horse, chicken, duck and pig.** The percentage of satellite cells was determined in primary muscle cultures by immunocytochemistry for the muscle-specific transcription factor Pax7. Representative fields of equine (*biceps femoris*) (**a**), chicken (*pectoralis major*) (**b**), duck (*pectoralis major*) (**c**) and porcine (*psoas major*) (**d**) cells at an early stage of differentiation are shown. Arrows show cells which were negative for Pax7. Haematoxylin counterstaining was used. Each species analysed presented a significantly different percentage of Pax7 positive cells (p < 0.001) (**e**) with chicken displaying the highest number (≈90%), followed by horse (≈80%), duck (≈70%) and pig (≈40%). The results were evaluated using a pairwise *t* test. Graph shows the mean percentage of Pax7 positive cells with standard deviation.

Current methods of isolation of muscle satellite cells from different animal species are highly variable making inter-species comparison problematic. Here, we have simplified a previous published method described to isolate muscle satellite cells in mice [21] and applied it to several agriculturally important avian and mammalian species: duck, chicken, horse, cow and pig. Following the revised method described in the Methods section, a high yield of muscle satellite cells was achieved for the species evaluated (horse, chicken, duck and pig) without the need for Percoll centrifugation or pre-plating. Muscle satellite cells could be identified by their expression of the muscle-specific transcription factor Pax7 [22]. To assess the relative purity of the isolated cells as muscle satellite cells immunocytochemistry for the detection of Pax7 was performed (Figure 1 a-d). A high percentage of isolated cells in all species (horse, chicken, duck and pig) expressed Pax7 (Figure 1 e). Note that Pax7 was readily detected in all the species examined except for bovine cells where we could not find a suitable antibody that cross-reacted with bovine. Each species analysed presented its own distinctively significant percentage of Pax7 positive cells (p < 0.001), with chicken displaying the highest number (≈90%), followed by horse (≈80%), duck (≈70%) and lastly, pig (≈40%).

**Figure 2:**
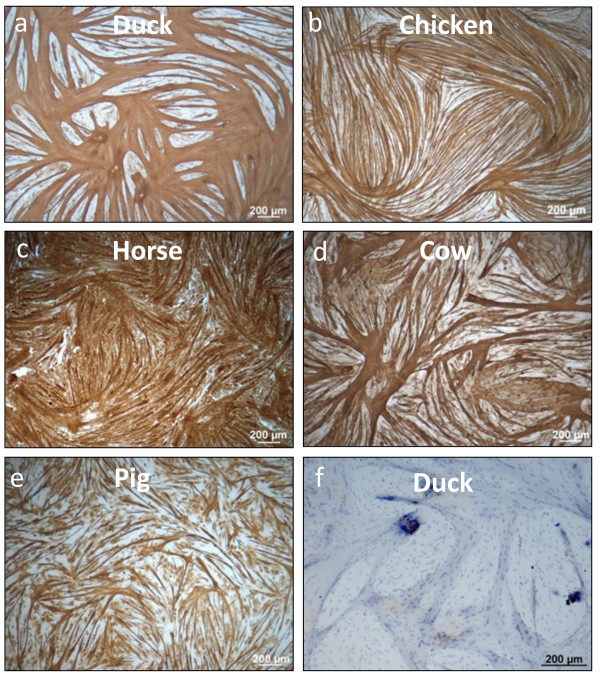
**Terminally differentiated primary muscle cultures from different animal species immunolabeled for the muscle-specific intermediate filament desmin.** Duck muscle (*pectoralis major*) cells were cultured for 4 days (**a**), chicken muscle (*pectoralis major*) cells for 6 days (**b**), equine muscle (*biceps femoris*) cells for 4 days (**c**), bovine muscle (*psoas major*) cells for 3 days (**d**), porcine muscle (*psoas major*) cells for 4 days (**e**). No primary antibody control duck muscle cells (**f**) was counterstained with Harris’ Haematoxylin. Note: days of culture are counted from seeding day 0.

**Figure 3:**
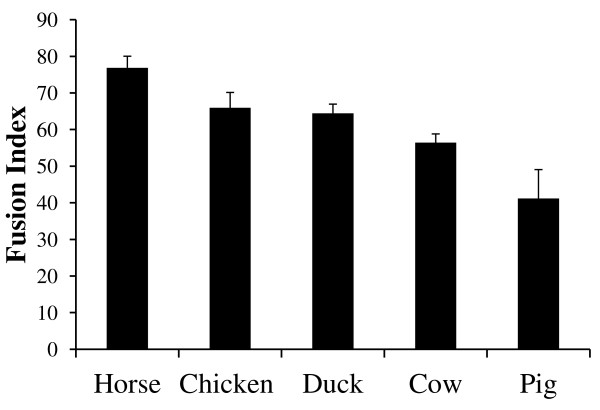
**Fusion index in terminally differentiated primary avian and mammalian muscle cultures.** Each species displayed a significantly different fusion index (p < 0.05) compared with other species, with the exception of the avian species (p > 0.7). Horse presented a significantly higher fusion index than all the species tested (p < 0.05). Remarkably, ≈77% of the equine cells fused into multi-nucleated myotubes. Porcine cells achieved the lowest level of fusion with ≈ 41% of the cells fusing into myotubes. The results were evaluated using a pairwise *t* test. Graph shows the mean fusion index with standard deviation.

In addition, fully multi-nucleated syncytia were achieved in our primary muscle cultures without overgrowth of non-myogenic cells as assessed by immunolabeling for the muscle-specific intermediate filament desmin (Figure 2). High fusion index was achieved for all the species, except the pig (Figure 3), using the same media for proliferation and differentiation. Each species exhibited a significantly different fusion index (p < 0.05) compared with other species, with the exception of chicken and duck (p > 0.7). Horse presented a significantly higher fusion index than all the species tested (p < 0.05) with ≈ 77% of the cells fused to become myotubes. This is an impressive fusion index considering that these are primary skeletal muscle cells not derived from clonal isolation. Fusion index of the avian species (≈65%) was significantly higher (p < 0.01) compared with that of cow (≈52%) and pig (≈41%). Pig showed the lowest but still substantial fusion index compared with the other species (p ≤ 0.01) with ≈ 41% of the cells forming myotubes. Porcine primary muscle cells isolated by more technically demanding methods (such as flow cytometry) have achieved a fusion index of ≈ 65% [23].

Key known factors which affect the isolation of satellite cells and/or modulate myogenesis *in vitro* are: (a) the extracellular matrix protein chosen to coat the culture plates [24,25], (b) the proteolytic enzyme(s) used to digest the muscle tissue [6], (c) the manner in which satellite cells are released from the myofibres -enzymatic digestion, mechanical dissociation (vigorous vortexing or triturations) or a combination of both [26,27], (d) the cell seeding density [28]- and (e) the choice of growth and differentiation culture media [11,13,29,30]. Taking into account all these factors, we modified Yablonka’s method [21] in the following aspects: (a) the extracellular matrix protein for coating the cell culture surface was changed from gelatin to type I collagen from rat tail, (b) the collection, digestion and culture media used were modified as described in Methods, (c) for simplicity, the batch of horse serum (HS) was not pre-selected for maximal fusion, (d) muscle tissue was enzymatically digested in a water bath without shaking, (e) the use of only 10-ml pipettes to triturate the muscle tissue, and (f) instead of using Millipore Swinney filters, the digested cell suspension was conveniently filtered through a 40-μm cell strainer. We chose to employ pronase as the proteolytic enzyme because of its ability to digest the basal membrane [6]. Pronase worked well in all species tested and has been extensively used by others to isolate satellite cells from different animal species [21,31-33].

After establishing the level of muscle satellite cells in freshly isolated cells, we performed immunocytochemistry to detect several muscle-specific muscle markers in terminally differentiated muscle cultures.

**Figure 4:**
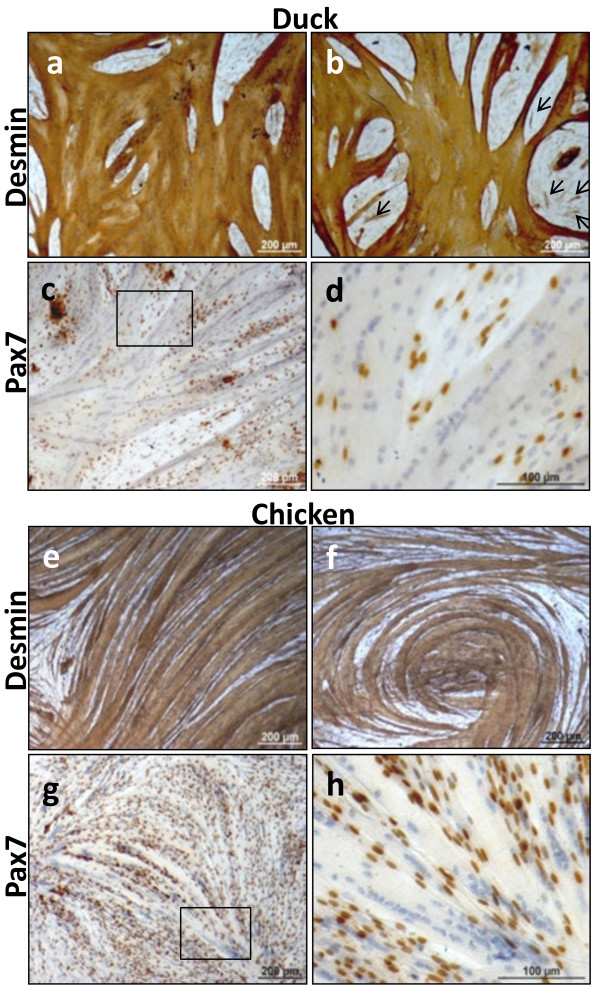
**Terminally differentiated primary duck and chicken muscle cultures immunolabeled for the muscle markers desmin and Pax7, counterstained with Harris’ Haematoxylin.** Duck breast muscle cells fused en masse to form large multi-nucleated syncytia. Extensive desmin presence (brown staining) in duck myotubes (**a, b**) with reduced detection of desmin confined to a small proportion of duck myoblasts (b, arrows). Duck myoblasts were identified by the expression of Pax7 in the myonuclei (brown staining) (**c, d**). Note the absence of Pax7 protein in myotubes. Chicken breast muscle cells achieved comparable level of fusion; myotubes in panel a and e each contained over 300 nuclei. Morphologically, fused chicken cells displayed more typically the myotubular structural shape (**e, f**). Chicken muscle cultures showed similar expression patterns of myogenic markers to duck cultures with desmin detection in the sarcoplasm of myotubes (**e, f**) and Pax7 protein in myoblast nuclei (**g, h**).

It has been previously reported that only a small proportion of chicken myoblasts express desmin in culture [34]. In the present study we also found little expression of desmin in chicken as well as duck myoblasts (Figure 4a, b, e and f). By contrast, desmin was extensively expressed in both chicken and duck myotubes (Figure 4a, b, e and f). During terminal differentiation, duck myoblasts were able to fuse en masse, forming very large multi-nucleated syncytia which often grew on top of replicating myoblasts. Pax7, an early muscle-specific paired-homeobox transcription factor, was readily detected in the nuclei of myoblasts but not in myotubes of chicken primary skeletal muscle cultures [31]. Likewise, we found Pax7 nuclear expression in duck myoblasts only and not in myotubes (Figure 4c and d). In our avian cultures, the pattern of desmin and Pax7 expression in muscle cells did not differ between duck (Figure 4a-d) and chicken muscle cells (Figure 4e-h). However, the two avian species showed distinctive myotube patterns. Duck myotubes were sheet-like whereas chicken myotubes were more muscle fibre-like with parallel fibres formation. In addition, after terminal differentiation duck myotubes would more readily contract and detach from the culture surface pointing to differences in the contractile ability of the newly formed chicken and duck myotubes.

**Figure 5:**
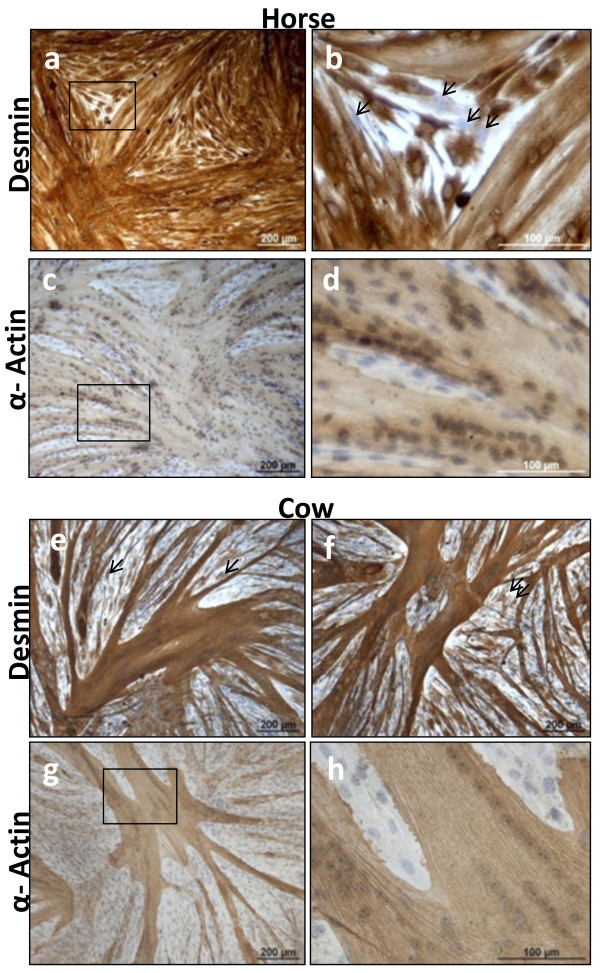
**Extensive differentiation and fusion of primary equine and bovine muscle immunolabeled for the muscle markers desmin and α-sarcomeric actin.** Equine muscle cells isolated from the red (oxidative) *biceps femoris* fused as readily as duck muscle cells (**a-d**). At day 4 of culture, equine cells appeared to express the highest level of desmin in relation to all the species examined (**a, b**). With over 95% of cells positive for desmin, the equine muscle culture ostensibly contained very few non-muscle cells (b, arrows). α-Sarcomeric actin immunolabeling (brown staining) was detected in equine myotubes but not in mononucleated cells (**c, d**). Bovine myogenic cells from *psoas major* (oxidative muscle type) also showed extensive cell fusion after 3 days of culture (e-h). Desmin expression was present in all myotubes (**e, f**) but also in some myoblasts (e arrows, f arrows). α-Sarcomeric actin was detected in bovine myotubes only (**g, h**). Haematoxylin nuclear counterstain was used.

Horse myogenicity, as indicated by the proportion of desmin positive cells in culture, appeared amongst the highest of all the species investigated. Over 95% of equine cultured cells were desmin-positive (Figure 5a and b). Equine muscle cells possessed very few contaminating cells, challenging the general notion that mammalian species have higher number of non-myogenic cells when compared with avian species [35]. Unlike the avian species, both equine myotubes and myoblasts strongly expressed desmin (Figure 5a and b), in agreement with previous observations [36]. Massive whorls of equine myotubes were evident when immunolabeled for α-sarcomeric actin (Figure 5c and d). Such was the extent of syncytia formation that the act of fixation with acetone:methanol was often sufficient to induce their detachment from the culture surface. Equine muscle cells were able to grow and differentiate with the same medium: Dulbecco’s Modified Eagle Media (DMEM)-Glutamax I and 20% fetal calf serum (FCS), without the need for serum depletion as previously documented [10,37].

Bovine muscle cells were also readily able to proliferate and differentiate without necessitating a specific differentiation medium which was required in previous reported primary bovine muscle cultures [11,29,38,39]. After 3 days of seeding in 24 well plates, bovine muscle cells underwent terminal differentiation and fusion into extensive and large multi-nucleated myotubes (Figure 5e-h). Desmin expression was predominantly found in myotubes but also in a small number of myoblasts (Figure 5e and f) as previously reported in bovine skeletal muscle cells [40]. Predictably, α-sarcomeric actin was restricted to myotubes only (Figure 5g and h).

**Figure 6:**
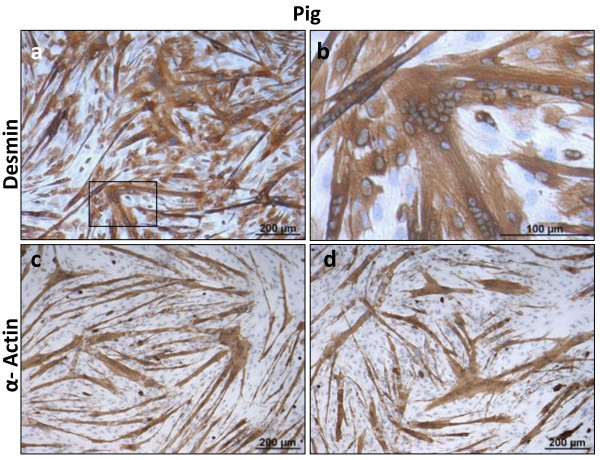
**Primary porcine muscle (*psoas major*) cells showed the lowest cell fusion rate out of all the species evaluated.** Satisfactory cell fusion was achieved at 72 h of culture with medium containing 0.4% Ultroser G. Cells fused en masse at small areas but predominantly fusion occurred in the form of thin myotubes. Desmin (brown staining) was detected in myotubes and some myoblasts (**a, b**). Note the high number of nuclei (>45) present in some myotubes (**b**). α-Sarcomeric actin staining (brown colour) was only found in terminally differentiated pig myotubes (**c, d**). Harris’ Haematoxylin nuclear counterstain was used.

Porcine skeletal muscle cells showed the lowest fusion rate in relation to the other avian and mammalian cells examined (Figure 2e). To establish culture conditions that promote better differentiation and fusion several types of media were evaluated, including (1) DMEM supplemented with 0.4% Ultroser G (Pall), (2) DMEM:Ham’s F12 supplemented with 1% insulin-transferrin-selenium (ITS) (Invitrogen) and (3) DMEM supplemented with 2% HS and 2 mM L-glutamine. Improved myotube formation was most evident using serum replacement 0.4% Ultroser G which is at a concentration equivalent to 2% FCS (Figures 2e and 6). Ultroser G has previously been documented to enhance myotube formation when compared with the commonly used serum-containing media in the culture of human skeletal muscle cells [30] and mouse C2C12 muscle cells [41]. Pig myogenicity was assessed by desmin and α-sarcomeric actin staining in cells differentiated with 0.4% Ultroser G for 72 h. A large proportion of porcine myoblasts expressed desmin in culture in accordance with previous studies [42]. Large multi-nucleated myotubes also expressed desmin (Figure 6a and b) which in conjunction with myoblasts, constituted approximately 80% desmin positive cells. α-Sarcomeric actin immunolabeling was limited to myotubes (Figure 6c and d). It should be stressed that only porcine cells required a serum reduction to achieve a high degree of fusion. Muscle cells from the other species proliferated and fused efficiently and rapidly in the presence of high serum concentrations (>10%).

**Figure 7:**
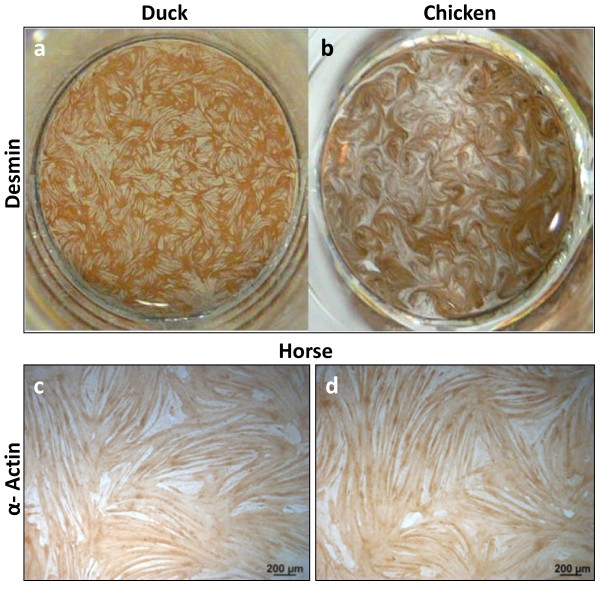
**Muscle cells from avian and equine species fused most extensively practically covering each culture well.** A 24-well plate showing desmin positive myotubes (brown colour) in duck (**a**) and chicken (**b**) cultures grown for 4 and 6 days respectively. More than 60% of the surface area was covered by terminally differentiated fused myotubes. α-Sarcomeric actin was localised to equine myotubes which almost completely covered the entire culture surface (**c, d**).

Out of all the species examined, muscle cells from duck (Figure 7a), chicken (Figure 7b) and horse (Figure 7c and d) fused most extensively covering most of each culture well. Duck and chicken muscle cultures exhibited highly myogenic characteristics which are also characteristic of turkey satellite cell cultures [9]. Bovine and porcine muscle cells occasionally formed large myotubes. The difference seen in the ability of fusion between different animal species was also noted in muscle cultures grown from the *fibularis longus* (red muscle) of chicken and duck, and the *longissimus doris* (white muscle) in pig (pictures not shown), suggesting that the observed degree of fusion is specific to the animal species. Primary muscle cells derived from the *fibularis longus* muscle of chicken formed parallel fibres while duck myotubes were sheet-like as previously observed in the *pectoralis major* of these species, indicating that both, the patterns and level of fusion, are a species-specific phenomenon.

In our study, equine and bovine muscle satellite cells were harvested from adult animals and appeared to result in reduced yield of satellite cells in relation to the other younger animal species. This apparent reduction in yield could be related to reduced satellite cell density in muscle tissue with age as observed in some species [43-45][46]. Nevertheless, once cells reached 70% confluence, both bovine and in particular equine satellite cells, differentiated and fused rapidly to form large multi-nucleated myotubes. Equine cells fused within a short period of several hours. Whether the proliferation and/or differentiation potential of satellite cells *in vitro*[45,47-49] and *in vivo*[50] are adversely affected by age remains to be fully resolved. Although we did not quantitatively monitor proliferation, our primary bovine and equine muscle cultures displayed an exceptional degree of myogenic differentiation, indicating that there is no need to exclusively use neonatal muscles to achieve satisfactory yield and myotube morphology *in vitro*.

## Conclusions

The described modified method represents a versatile and robust way to isolate satellite cells from a variety of animal species. This relatively simple method achieves good yield of high quality satellite cells that are demonstrably able to proliferate, differentiate and fuse into extensive myotubes.

## Methods

### Preparation of primary muscle cultures

Skeletal muscle samples were derived from several 4- to 6-week-old Pekin ducks, from Cherry Valley Farms UK, several 4- to 6-week-old broiler chickens (ROSS 308 strain), from PD Hooks Hatcheries Thirsk UK, a 7- to 8-year-old thoroughbred horse, obtained at post mortem, several 5- 8-week-old Landrace-Large White hybrid pigs, and several 18- to 20-month-old Limousine cross heifers obtained from a local abattoir. All animals were euthanased following approved humane methods. *Pectoralis major* (breast) muscle from chicken and duck, *biceps femoris* from horse and *psoas major* from pig and cow were aseptically removed. Each muscle was washed once in phosphate buffered saline (PBS) and placed in collection medium, high glucose Dulbecco’s Modified Eagle Media (DMEM-Glutamax I) (Invitrogen) supplemented with 1% penicillin and streptomycin (P/S) and 1% amphotericin B. All media and PBS were used at room temperature.

In the cell culture hood, each muscle tissue was washed several times in PBS, followed by the removal of associated connective tissues with a sterile scalpel before mincing with scissors. The procedure for the isolation of muscle satellite cells was modified from a previously described method as detailed by Yablonka [21] and described below.

#### Proteolytic digestion of muscle tissue

Minced muscle was washed once with collection medium. Approximately 4 g of minced muscle were incubated at 37 °C for 1 hour in a 50-ml conical tube in a water bath with 30 ml of dissociation medium, DMEM:Ham’s F12 containing 15 mM HEPES (Invitrogen), supplemented with pronase (Sigma) at 1.4 mg/ml, 1% P/S and 1% amphotericin B. The digested muscle tissue was collected by low speed centrifugation for 6 min at 300 *g* and washed once in PBS (~10 ml per tube).

#### Isolation of muscle satellite cells

Digested muscle pellet from the previous step was resuspended in 15 ml of collection medium and subjected to vigorous triturations (repeated pipetting of tissue suspension with a 10-ml pipette for approximately 15 times). The suspension was subjected to low speed centrifugation to sediment the tissue fragments while the cells remained in suspension. The supernatant containing the released cells was collected and filtered through a 40-μm cell strainer (BD Falcon) into a new 50-ml conical tube to remove other cell debris. The flow through was then diluted in PBS (~40 ml) and centrifuged for 10 min at 800 *g* to pellet the cells.

#### Plating of muscle satellite cells

The washed cell pellet obtained in the previous step was re-suspended in appropriate growth medium and directly plated onto T75 flasks which had previously been coated with 1% type I collagen from rat tail diluted in sterile water (Sigma). All flasks were rinsed with PBS before use. Erythrocytes in the cell preparation could make an accurate cell count difficult, especially with red (oxidative) skeletal muscle. Empirically each cell pellet obtained from 4 g of minced muscle tissue would yield sufficient cells to seed onto one T75 flask. We observed a decrease in plating efficiency when more tissue was digested and plated onto one T75 as the amount of debris appeared to interfere with the attachment of cells [51]. All cells in this study were subjected to a maximum of 2 passages (trypsinization) and maintained at 37 °C in the presence of 5% CO_2_. For the first two days after isolation, cells were washed several times with warm PBS to remove tissue debris and erythrocytes; during this period 1% amphotericin B was also added to the growth medium. Cells were allowed to proliferate for 2 days in avian and porcine cultures and 4 days in equine and bovine cultures before trypsinization into 24-well collagen coated plates (approximately 20,000 cells per well) for immunocytochemistry. The remaining cells were frozen using 90% horse serum (HS) and 10% dimethyl sulfoxide and stored in liquid nitrogen.

#### Composition of Growth (GM) and differentiation medium (DM)

Avian cells were grown as well as differentiated in DMEM-Glutamax I (high glucose) with 10% HS, 4% chick embryo extract (Egg Technologies) and 1% P/S. The batch of HS was not pre-selected for maximal fusion. Equine and bovine cells were grown and differentiated in DMEM-Glutamax I (high glucose) supplemented with 20% fetal calf serum (FCS) and 1% P/S. Pig cells were grown using skeletal muscle basal medium-2 (SkBM-2) supplemented with the SkBM-2 SingleQuots kit (Lonza); when they reached 90% confluence, the GM was replaced with DM (DMEM containing 0.4% Ultroser G (Pall) and 1% P/S).

### Cell fixation and immunocytochemistry

Cells were fixed at room temperature with acetone-methanol (1:1) for 10 min for immunochemical staining for muscle cell markers. Acetone:methanol solution was added at room temperature. Fixed cells were washed with Tris buffered saline (TBS) and immunochemical staining was carried out using EnVision + system-HPR (DAB) kit (DAKO) following the manufacturer’s instructions. In brief, fixed cells were incubated for 10 min with an endogenous peroxidase blocker. Cells were then washed once with TBS and were incubated with a 10 μg/ml concentration of primary mouse antibody against Pax7 (R&D systems) (for avian and equine cells), or a mouse antibody against Pax7 (Developmental Studies Hybridoma Bank, kindly provided by Dr. Dylan Sweetman) used at 1:50 dilution (for porcine cells), or a rabbit anti-desmin antibody (Abcam) at 5 μg/ml or a mouse anti-α-sarcomeric actin (clone Sr-1) (Dako) at 1:50 dilution for 40 min at room temperature. Cells were rinsed four times with generous volumes of TBS and were incubated with an anti mouse or anti rabbit HRP labelled polymer for 40 min. After rinsing four times with TBS, cells were incubated with DAB substrate for 6 min, washed with TBS and counterstained with Harris’ Haematoxylin. Negative controls were performed omitting the use of primary antibody.

### Percentage of Pax7 positive cells in freshly isolated cells

To determine the percentage of Pax7 positive cells, immunocytochemical labeling for Pax7 was carried out. After counterstaining with Harris’ Haematoxylin, 10 arbitrary fields were photographed using a x40 objective and cells presenting positive labeling were counted with respect to the total cell number. Approximately 1,000 to 1,500 total cells were counted for each species.

### Fusion index

To compare the fusion rate achieved by the different species tested, fusion index was determined in differentiated muscle cell cultures. For this purpose, after either desmin or α-sarcomeric actin immunocytochemical labeling followed by counterstaining with Harris’ Haematoxylin, 3 to 4 random fields were photographed using the 10-time objective lens. Nuclei within myotubes and nuclei within unfused cells were counted manually and the number of nuclei in myotubes was expressed against the total number of nuclei as a percentage. Approximately 5,000 to 7,000 cells were counted for each species. Only myotubes containing at least three nuclei were scored.

## Competing interests

The authors declare no competing interests.

## Authors’ contributions

BBP grew the primary muscle cultures, performed the immunocytochemistry labeling, the cell counts and wrote the first draft. SVK and KCC edited the draft. RKN helped to carry out the Haematoxylin counterstaining and participated in the design of the study. All authors read and approved the final manuscript.
